# Why are hospital doctors not referring to Consultation-Liaison Psychiatry? – a systemic review

**DOI:** 10.1186/s12888-016-1100-6

**Published:** 2016-11-09

**Authors:** Kai Yang Chen, Rebecca Evans, Sarah Larkins

**Affiliations:** 1 James Cook University, 1 James Cook Drive, Townsville, QLD 4811 Australia; 2 Townsville Hospital and Health Service, 100 Angus Smith Drive, Townsville, QLD 4814 Australia

**Keywords:** Consultation-Liaison Psychiatry, Hospital psychiatry, Barriers to referral, Consultation inpatient

## Abstract

**Background:**

Consultation-Liaison Psychiatry (CLP) is a subspecialty of psychiatry that provides care to inpatients under non-psychiatric care. Despite evidence of benefits of CLP for inpatients with psychiatric comorbidities, referral rates from hospital doctors remain low. This review aims to understand barriers to CLP inpatient referral as described in the literature.

**Methods:**

We searched on Medline, PsychINFO, CINAHL and SCOPUS, using MESH and the following keywords: 1) Consultation-Liaison Psychiatry, Consultation Liaison Psychiatry, Consultation Psychiatry, Liaison Psychiatry, Hospital Psychiatry, Psychosomatic Medicine, the 2) Referral, Consultation, Consultancy and 3) Inpatient, Hospitalized patient, Hospitalized patient. We considered papers published between 1 Jan 1965 and 30 Sep 2015 and all articles written in English that contribute to understanding of barriers to CLP referral were included.

**Results:**

Thirty-five eligible articles were found and they were grouped thematically into three categories: (1) Systemic factors; (2) Referrer factors; (3) Patient factors. Systemic factors that improves referrals include a dedicated CLP service, active CLP consultant and collaborative screening of patients. Referrer factors that increases referrals include doctors of internal medicine specialty and comfortable with CLP. Patients more likely to be referred tend to be young, has psychiatric history, live in an urban setting or has functional psychosis.

**Conclusion:**

This is the first systematic review that examines factors that influence CLP inpatient referrals. Although there is research in this area, it is of limited quality. Education could be provided to hospital doctors to better recognise mental illness. Collaborative screening of vulnerable groups could prevent inpatients from missing out on psychiatric care. CLP clinicians should use the knowledge gained in this review to provide quality engagement with referrers.

## Background

In 2012, The Australian Institute of Health and Welfare reported that people with comorbidity of any mental health and physical illness were significantly more likely to be hospitalised than people with only a mental health condition, or only a physical illness [1]. The prevalence of mental illness among hospital inpatients ranged from 26.1 to 38.7 % [2–5]. Among the mental illnesses found among inpatients, prevalence of depression varied from 5.1 to 33.5 % [2–10] and anxiety disorders were estimated around 5.8 % [2].

Hospital inpatients with any psychiatric comorbidity are more likely to utilise health care resources than those with only medical conditions. Levenson and colleagues found that patients with psychopathology or pain had longer hospital stays, more procedures performed and incurred more hospital charges [11]. Saravay and associates demonstrated in a prospective study that severity of the psychiatric comorbidity was associated with the length of stay in hospital [12]. Patients with cognitive impairment also have increased length of stay [13–15].

Consultation-Liaison Psychiatry (CLP) may help to improve outcomes for inpatients with psychiatric comorbidities. CLP is defined as a subspecialty of psychiatry that provides psychiatric education and care to non-psychiatric departments of a general hospital [16–18]. CLP may also provide psychiatric clinical care to patients in primary care settings [19]. The aim of CLP is to address the mental health needs of patients who are being treated in a non-psychiatric setting.

Involvement of CLP had been shown to improve outcomes in several subsets of inpatients. In fact, elderly patients with a fractured femur, with liaison psychiatrist input, were twice as likely to be discharged home and had a shorter length of stay, compared to patients with no psychiatric involvement [20]. Desan and his colleagues found that psychiatric consultation reduces length of stay for medical inpatients [21]. Furthermore, Cassem and Hackett showed that coronary care patients who were referred for psychiatric consultation were three times less likely to die compared to the rest of the coronary care unit [22].

Despite mounting evidence supporting the involvement of CLP for inpatients with psychiatric comorbidities, referral rates from treating doctors remain low at 0.72 to 5.8 % [23–29].

This study aims to present a systematic review on barriers to referral to CLP in the hospital or inpatient setting.

## Methods

This review systematically identified the relevant literature using predefined search and inclusion strategies per MOOSE guidelines.

A systematic search was conducted through electronic databases including MEDLINE, PsychINFO, CINAHL and SCOPUS for articles published between 1 January 1965 and 30 September 2015.

Search terms generated were used in all different variants, singular or plural forms and included MESH and free text terms. These included: (1) Consultation-Liaison Psychiatry, Consultation Liaison Psychiatry, Consultation Psychiatry, Liaison Psychiatry, Hospital Psychiatry, Psychosomatic Medicine, (2) Referral, Consultation, Consultancy and (3) Inpatient, Hospitalised patient, Hospitalized patient. Articles that included all the above search terms (1 and 2 and 3) in abstract or title were screened.

Bibliographic screening of included articles was also performed to identify further articles.

Inclusion criteria for articles included: (1) published in English language, and (2) contributes to understanding barriers to CLP referral.

Exclusion criteria for articles included: (1) Non-English publication, (2) Non-human trials, or (3) Non-adult population, or (4) Did not contribute to furthering understanding of barriers to CLP referral.

## Results

**Fig. 1:**
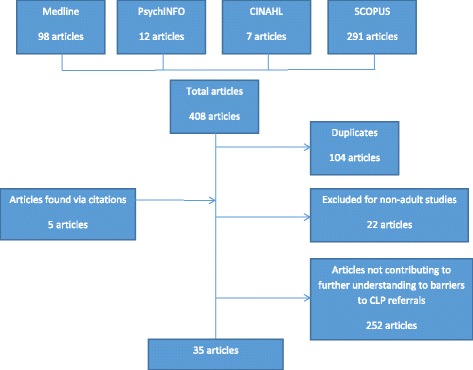
Selection of articles by search strategy

The process in which articles were found and excluded is summarised in Fig. 1.

A summary table of the thirty-five articles included for analysis can be found in Appendix. A thematic review of the barriers and enablers of CLP referrals from these 35 articles was conducted. We grouped these factors into three categories: (1) Systemic factors; (2) Referrer factors; (3) Patient factors. For each category’s item, we gave an indication of whether it has an influence to increase or decrease CLP referral.

### Systemic factors

Systemic factors are defined as the environment factors (external to the hospital doctors or the patients). The systemic factors that influence CLP referrals are summarized in Table 1.

**Table 1:** Systemic factors possibly influencing referrals to CLP

Systemic factors	
Increase CLP referral	1. Presence of dedicated CLP Service [30–33, 35] 2. Active engagement of CLP consultant [35] 3. Collaborative screening of inpatients [34]
Decrease CLP referral	1. Lack of detailed suicide prevention strategy [36, 37] 2. Poor CLP communication [32]
Unclear influence	1. Work pressure [38] 2. Presence of mental health nurse [39]

In a naturalistic longitudinal study based in Glasgow, Brown and his colleagues took advantage of the introduction of a new resident CLP team and studied the referral patterns over a period of 7 years [30]. The psychiatric needs of the hospital were previously met by psychiatrists from an associated psychiatric hospital and not a dedicated CLP team. It was found that referral rates increased over the study period, with a disproportionate increase in referrals of inpatients who were not involved in acts of deliberate self-harm. They suggested that the increase in referrals were due to the presence of a dedicated CLP unit. These results were replicated in one other similar study that looked at referral rates after introduction of a CLP unit [31]. Accessibility to a CLP service was also mentioned in several surveys [32, 33] as a factor that may increase CLP referrals.

The presence of a dedicated CLP team may not be adequate. Collaborative screening of inpatients with their treating team can further increase referral rates. In a Swiss study, twice weekly multidisciplinary meetings were held on a medical ward, involving psychiatrist, medical consultant and nurses. It was found that referral rates increased from 4 to 32 % when collaborative screening of patients was done [34]. In addition, active engagement of medical teams by CLP consultant is suggested to increase referrals in a one year single-site German observational study [35], although active engagement was not clearly defined.

Lacking a strategy towards management of psychiatric patients may contribute to low referral rates. It was found that past suicide attempts were not correlated with psychiatric referral in South Korean hospitals [36]. Authors of this study attributed this to a lack of strategies for detailed suicide prevention in Korean emergency departments and possible prejudice towards psychiatric consultation. This was supported by the fact that studies in countries other than South Korea had found suicide attempts to be predictive of referrals [37].

Limited work hours were suggested to be impacting on referrals to CLP. Caplan and his team suggested that there may be increased tendency by referrers to outsource the building of doctor- patient relationships to CLP [38]. The impact of work pressure may decrease referral rates by limiting the referrer’s time or resources to manage a CLP referral. It cannot be concluded at this time if work pressure affects referral as there were no studies looking at correlation of workload of referrer and referrals to CLP.

When close collaborative work with a psychiatrist is not possible, liaison work by mental health nurses may increase referral rates. There were no studies that considered the association of referral rates with the presence of a mental health nurse. One article that described the experience of mental health nurse liaison in a head-and-neck cancer unit, reported some subjective benefits [39]. The referral patterns were not clear in this study, but the authors suggested that referral rates were more dependent on the cancer unit staff on-shift, rather than presence of mental health nurse. Further elaboration of how referral is more associated with some ward staff was not done in this report.

### Referrer factors

Referrer factors are factors that are associated with the characteristics of the practitioners who may potentially utilise CLP services by referring their hospital patients. These factors are summarised below (Table 2).

**Table 2:** Referrer factors possibly influencing referrals to CLP

Referrer factors	
Increase CLP referral	1. Internal medicine speciality [33, 40, 52–55] 2. Positive attitude towards CLP [35, 41] 3. Discomfort in competency assessment and management [57]
Decrease CLP referral	1. Young age [33] 2. Stigma [32, 33] 3. Belief that other mental health professionals may do equally well [32] 4. Patient preference [33] 5. Poor rapport with psychiatrist [33] 6. Belief that referrer can manage without psychiatric help [40] 7. Poor recognition of mental illness [33, 42–51, 54]
Unclear influence	1. Different expectation of CLP service delivery [56]

Few studies attempted to investigate barriers to CLP referral from the referrer’s perspective. In 1990, Thompson and associates found through a survey of 200 hospital doctors (response rate of 35 %), that physicians agreed upon three main reasons for not referring for psychiatric consultations [32]. These three reasons were stigma, poor communication from psychiatrists and a belief that other mental health professionals may do equally well for the patient's health at reduced cost. It was difficult to assess the quality of this study given the process for survey development and validation (if conducted) was not outlined. No statistical analysis of data was reported.

In 1971, Mezey and Kellett conducted a survey with 106 hospital consultants using a modified survey previously used with general practitioners in 1966 [33]. Patients’ preference not to be referred was identified as the most common barrier to psychiatric referral. Stigma was the second most common reason, followed closely by poor access to services, and inadequate rapport with psychiatrists. Mezey and Kellett also analysed the demographics of the surveyed participants and suggested that older consultants were less likely to recognise psychiatric comorbidities in their patients, while younger consultants were more likely to have inadequate rapport with the psychiatrist. Surgeons and gynaecologists were less likely to refer than their physician counter-parts. Mezey and Kellett were unable to explain this difference between specialties in their survey.

In 1982, through a survey of 400 hospital doctors (including physicians and surgeons) in North Carolina Memorial Hospital, Cohen-Cole and Friedman realised that hospital doctors do not refer most of their patients identified with significant psychological issues [40]. In the same survey, 78 % of hospital doctors felt comfortable handling these psychological issues without psychiatry consultation. In addition, Cohen-Cole and Friedman found that attending physicians in internal medicine (62 %) and family medicine (43 %) tend to estimate more psychological components affecting their patients compared to surgeons (30 %), obstetricians-gynaecologist (19 %) and paediatricians (29 %).

The positive attitude of the hospital doctors towards a CLP service may increase referrals. In a 2015 survey by Hamdieh and team based in Iran, hospital doctors who had positive attitude towards their CLP service were more comfortable in making psychiatric referrals [41]. This result was supported by a one year single-site study of a German CLP service. Following one year of active engagement by the same CLP consultant, it was suggested that referrers were more comfortable with a psychiatric approach with their patients, resulting in more referrals [35].

While there might be differences in recognition of mental illness between specialties, a methodologically sound meta-analysis of 36 prevalence studies suggests that overall recognition of depression by non-psychiatric hospital doctors is lacking [42]. Overall sensitivity was found to be 36.4 % and specificity was 83.7 %. In 1995, in a prospective study of 987 medical and surgical patients in Monash Medical Center, Clarke and colleagues found diagnostic concordance of depression by referring doctor compared to a consultant psychiatrist was at 74 % [43] with a false-positive rate of 41 % and a false-negative rate of 15 %. This finding was replicated in several other studies [44–48]. Judd et al. found slightly better concordance of depression diagnosis in HIV patients (79 %) with false-positive rate of 20 % and false-negative rate of 23 %. Dilts et al. found low accuracy only in depression diagnosis but not cognitive impairment or substance use disorder. This difference may be due to hospital doctors finding difficulty in identifying clinical depression in physically unwell patients [44]. Canuto and colleagues suggest concordance in diagnosis of clinical depression between psychiatrist and other doctors increases with severity of depression and younger age [49]. Depression is, however not the only mental illness poorly recognised by hospital doctors. Drug and alcohol issues were also frequently missed by hospital doctors [50], although anxiety and psychotic disorders were found to have the lowest diagnostic concordance in a 5 year study [51].

There are differences in estimates of mental illness prevalence among hospital doctors of different specialties. Mezey and Kellet’s survey revealed that surgeons and gynaecologists were less likely to refer than their medical physician counterparts [33]. This was supported by reviews of referrals by surgical, obstetric and gynaecological doctors in several studies [52, 53]. Collating findings of Cohen-Cole and Friedman’s study with Mezey and Kellet’s, lower referral rates from surgeons, obstetricians and gynaecologists may be a result of poorer recognition of psychiatric issues. This view was supported by a study by Balestrieri et al. in medical and surgical inpatients in 2002 [54].

One may argue that lower referral rates from non-physician doctors may be linked to patients being treated adequately, without the need for referral. This was not found to be the case by Fauman in his survey of 11 hospital doctors. Findings suggested a significantly larger proportion of physicians willing to treat psychiatric disorders than their surgical or obstetric-gynaecological peers [55].

The difference in referral rates between specialties could also be accounted for by differences in expectations. In a questionnaire to 77 hospital doctors, De-Nour found that physicians tend to expect liaison service where psychiatrists participate actively in case conferences and routine management of patients [56]. This was in contrast to surgeons who expect a consultation service, where a psychiatrist advises on psychiatric management and diagnosis.

While the focus had been on presence of mental illness in patients, when assessment of competency was involved, many factors could increase referrals. Jourdan and Glickman found that over 25 % of patients referred to their CLP service were for assessment of competency [57]. This same group of patients had no mental illness and three quarters of them were found to be competent. Jourdan and Glickman explained that the fear and anxiety of doctors about medico-legal consequences, and poor understanding of management when patients refuse treatment were possible reasons for increased referrals.

### Patient factors

The presence of a psychiatric history increases the likelihood of referral. Fenichel and Murphy examined the decision-making process around making a psychiatric referral in the emergency department [58]. It was found that non-psychiatric staff often based their decision of referral on past psychiatry history if the patient presents with mild to moderate symptoms. In a study by Pritchard in 1972, patients with a psychiatric history are three times more likely to be referred for psychiatric consultation. However, Pritchard suggested that if the patient had previous contact with the psychiatric team in the same hospital, they might not be referred to CLP [37]. This was supported by lowered referral rates for patients with previous psychiatric contact in same hospital but did not reach statistical significance (Table 3).

**Table 3:** Patient factors possibly influencing referrals to CLP

Patient factors	
Increase CLP referral	1. Past psychiatric history [58] 2. Young age [37, 59, 60] 3. Urban setting [59] 4. Functional psychosis [37]
Decrease CLP referral	1. Organic psychosis [37] 2. Previous psychiatric contact at same hospital [37]
Unclear influence	1. Personality disorder (earlier referral) [62] 2. Depression (delayed referral) [62] 3. Race and socio-economic status [63, 64] 4. Stigma [65]

Referral is more likely to happen if the patient is of younger age. Marcus and team looked at data from 327 American hospitals and found that age was inversely correlated with psychiatric referral rates [59]. Other studies have supported the inverse correlation of age to referrals [37, 60, 61]. The same study from Marcus et al. did not find gender of patients to be useful as predictors to psychiatric referrals [59]. However, patients in urban settings were more likely to be referred. This was attributed to a significantly higher psychiatrist-to-population ratio in urban regions [59].

The psychiatric diagnosis of the patient may predict likelihood of CLP referral. Patients with functional psychotic diagnoses (including schizophrenia and psychotic depression) were more likely to be referred to CLP [37]. Patients with psychosis from organic causes (such as dementia and delirium) were less likely to be referred [37]. In a prospective study of 712 referrals over a five-year period examining timing of referrals, presence of personality disorder was found to predict earlier referral. This was in contrast to presence of depression where a delayed referral was more likely [62]. Although timing of the referral does not inform us of whether a referral has been made, one may expect previous poor recognition of depression from a non-psychiatrist as discussed earlier to have delayed and prevented referrals.

Patient’s race and their socio-economic status may influence psychiatric referrals. In a 1982 review of CLP referrals in a major teaching hospital, Craig found that white patients were more likely to be referred than non-white patients when an active liaison service was present [63]. However, once referred, services rendered to all patients by CLP were of no difference. Low referral rates exist similarly across all races if an active liaison service was not present. Craig suggested that this association may be as a result of higher emotional distress in lower socio-economic groups, which was unfortunately associated with non-white patients presenting to this teaching hospital. Collins and colleagues looked at referral patterns among different ethnic-cultural groups in San Diego Medical Center [64]. They found lower referral rates for Hispanics compared to other groups (Anglos, Blacks and Asians). Different ethnic groups were also more likely to be referred for different conditions. For example, requests for evaluation of depression and suicides were higher in Hispanics, and much reduced in Blacks. The authors were not able to draw conclusions from these results and suggested more research into cultural factors that may influence manifestations of poor mental health and CLP referrals.

The perceived stigma by hospital doctors identified by Mezey and Kellet may not be present from the patient’s perspective. Klein and team interviewed 48 medical inpatients and found that 81 % were either moderately to very agreeable to having a psychiatric consultation [65].

## Discussion

This review is the first to closely examine the possible factors reported in the literature that could influence CLP inpatient referrals. These studies were from diverse health care systems in different areas of the world. Of the thirty-three articles that stated their country of origin, thirteen of the studies originate from the US, six were from UK, five were from Europe other than UK (Germany, Ireland, Italy and Switzerland), four were from Australia and five were from Asia (Japan, Iran, Israel, South Korea, and Taiwan). There is a lack of research in this area from South America and Africa, which may reflect the lack of prioritisation for CLP services or research in these continents.

It is difficult to comment on hospital factors that might influence the generalisability of this review. However, factors such as hospital type (e.g., tertiary referral, specialist, regional); service population or catchment area; and level of non-CLP staff training may affect the application of these findings in various settings. While some studies in this review did describe inclusion of non-tertiary/tertiary, regional, private or geriatric hospitals, others were not clear in their description. We would expect CLP service utilisation by hospital doctors to be different depending on their service population and location. Therefore, the review reflected a global perspective of the literature available and may not apply to individual health systems.

Despite a review of the past fifty years, only thirty-five studies were included. This may reflect lack of research into direct factors influencing CLP referrals. Lack of rigorous research into this topic could also explain the paucity of research included in this review. For example, a significant number of studies reported cross-sectional referral patterns of their respective CLP hospital service. These studies provided no new perspective and did not offer explanations for referral barriers.

Most of the articles found in this review were of low methodological quality, mainly comprising of surveys or retrospective chart reviews. The mix of studies included sixteen retrospective chart reviews, eight surveys, seven prospective studies, two reports of subjective experiences, one epidemiological study, and one meta-analysis. Only seven of the articles were published in the last ten years, suggesting the lack of recent research. Most of the surveys looking at the referrers’ perspectives were published from 1970s to 1980s. This could reflect the early struggle of CLP to improve engagement with other medical specialties. Modern CLP services may not require direct patient referrals from hospital settings. The availability of outpatient CLP services may lower inpatient referral rates but increase overall number of CLP referrals.

Studies using surveys did not explain how individual items on the surveys were formulated. It was possible that survey items may reflect potential confirmation and selection bias from study authors. Validation data was rarely presented, so there may also be validity issues; where survey responses did not accurately reflect the perspectives of hospital doctors. No qualitative study has yet been performed to investigate the viewpoints of referrers and users of CLP services. This may represent an important knowledge gap for future research.

It is acknowledged that the literature search was performed by only the first author and selection bias in identification of articles is possible. However, the purpose of the review was to understand any factors published in the literature that could influence CLP referrals. Selection of articles was based on stated criteria but erred on the side of being over-inclusive. Missing articles, if any, were more likely due to human error.

The challenge for increasing psychiatric referrals may be quite similar in the primary care setting. Several inpatient barriers to psychiatric referral echoed barriers found in primary care, such as availability of psychiatrist [66], time pressure [66], poor communication with psychiatrist [67, 68] and poor recognition of mental illness by primary care physicians [69, 70]. There may be considerable opportunity for future research that would be applicable to both inpatient CLP and primary care settings.

Comparing referral rates to prevalence of mental illness among hospital inpatients, it is clear that most inpatients with psychiatric comorbidities do not get referred by their treating team. The systemic factors found in the review suggested that quality of engagement is likely to influence referral rates [30–35]. Quality of engagement may involve a more active and communicative CLP service whose presence is clearly felt by the referrers. It may also involve building good working relationship with the referring team. If referrers feel that CLP consultation could bring benefits, they are more likely to continue referring. Many CLP services recognise this and use referrer satisfaction as an outcome measure for performance [71].

Quality of engagement may improve through means other than CLP service delivery. For example, research collaboration, outpatient CLP work and collaborative/integrated care with CLP are some methods to improve engagement with other specialties [72, 73]. There were no studies investigating the direct influence of these methods on inpatient CLP referrals rates. This is understandable given the complexity of confounders in hospital systems, complicating any potential research into the area.

Placement of mental health nurses in a liaison role may be beneficial. Allied health professionals such as social workers and nurses may spend more time with patients. There is some evidence that with training and adequate staffing, nurses may improve on detection of mental illness [74]. Liaison work using CLP allied health professionals may pick up referrals missed by hospital doctors. Future research could investigate the impact of CLP allied health professionals on referral rates.

While a lack of hospital protocols or policies to guide CLP referrals may contribute to poor referral rates, systemic strategies that seek to increase referrals may bring unintended consequences. Introduction of any strategies should be considered with care. For example, mandatory CLP referrals for inpatients with psychiatric comorbidities, could strain poorly resourced CLP teams. This may lead to poor rapport with referrers and decrease quality of engagement. This strategy would also depend heavily on the referrer’s ability to recognise mental illness, which had been shown to be lacking [33, 42–51, 54].

In terms of referrer factors, the review showed an at-risk group of hospital doctors who were less likely to refer their patients. These doctors include those of surgery and obstetrics-gynaecology subspecialties. Young, pre-specialist certified doctors were also less likely to make CLP referral. Considerations could be made to increase education, collaboration and communication to these at-risk doctor groups to increase CLP referrals.

Hospital doctor’s ability to recognise and diagnose psychiatric conditions affects referral rate. In support of this, an exploratory study by Shortell and Daniels looked at internists in private practice and their psychiatric referrals [75]. They found that internists who were qualified specialist, older and had more years in practice are more likely to refer their patients. Higher referral rates may also come from experienced doctors who were more apt at recognising mental illness.

In contrast, younger doctors who were not yet qualified specialist and had spent less time in practice may be referring fewer patients [33, 75]. A positive association may exist between self-perceived abilities to manage psychiatric issues and low referral rates. It was uncertain if young doctors' self-perceived ability to manage psychiatric conditions were reflective of their true capabilities. Further studies could shed light on this issue.

Stigma and patient preference were often raised as referral barriers by hospital doctors. As illustrated by Klein’s study on medical inpatients, patients often do not hold the same view as their doctors [65]. It would be important for the education of hospital doctors, so that referrals were not obstructed by their own perception of mental illness stigma.

Younger patients with functional psychosis were more likely to be referred. This implied that other patient groups such as geriatric population or patients with delirium or dementia may be neglected. Although there is an increasing demand for CLP services by the geriatric population in recent years [76, 77], these patients are at risk of missing on psychiatric care.

Two studies have investigated the impact of race and ethnicity on CLP referrals. While statistically significant differences were found, these studies were of an epidemiological nature that could only suggest an association rather than direct causality. Socio-economic status may be confounding the results and authors of these two studies were careful not to draw early conclusions. The impact of racial profiling by hospital doctors may be important for CLP services. Patients, regardless of their ethnicity or socio-economic status, should not be disadvantaged and be denied mental health care while hospitalised. Education of hospital doctors could correct this issue.

The review highlighted several patient groups that may be at risk. Collaborative screening of these vulnerable inpatient groups may be beneficial in providing fair mental health care.

## Conclusion

CLP presents an opportunity to improve health outcomes for inpatients and reduce burden on the health care system, but data shows that this service is currently underutilised. Understanding the potential barriers to CLP referral is an important first step in improving referral rates. Although there is research in this area, it is of limited quality. There is no qualitative research from referrers’ perspective, though such research may improve understanding of barriers to CLP referrals in the future. Education could be provided to at-risk hospital doctors to better recognise mental illness in their patients. Collaborative screening of vulnerable groups could prevent inpatients from missing out on psychiatric care. CLP clinicians should use the knowledge gained from this review to encourage quality engagement with referrers.

## Appendix

**Table 4:** Summary of articles included, details of the included articles including country, participants, study design and findings

Author/s, Date (In order of appearance)	Country, Setting	Participants (type and number)	Study design	Findings
Brown & Cooper 1987 [30]	UK, general hospital	1,140 inpatient referrals	Retrospective review of referrals in 1973, 1976, 1979	Dedicated CLP service increases referrals.
McCartney et al. 1989 [31]	US, university hospital	11,713 gynaecologic oncology and other cancer patients	Retrospective review of referrals, before and after introduction of gynaecologic oncology liaison program	Introduction of CLP program increases referrals
Thompson et al. 1990 [32]	US, Colorado	200 non-psychiatrist doctors (35 % response rate)	16 item survey	Most common reasons for not referring: Other mental health practitioners do just as well, lack of communication by psychiatrist, stigma, accessibility
Mezey & Kellett 1971 [33]	UK, London	106 consultants from 6 hospitals (83 % response rate)	10 item survey	Most common reasons for not referring: Patient’s preference, stigma, accessibility, poor rapport with psychiatrist. Surgery, Obstetrics and Gynaecology subspecialty associated with less referrals
Diefenbacher 2001 [35]	Germany, Berlin, Rudolf Virchow Hospital	208 inpatient referrals	Observational study of referral patterns over 1 year following introduction of CLP	Increase in referrals from medical and surgical wards. Decrease in urgent referrals, suggesting increased tolerance towards psychiatric conditions.
Camus et al. 2003 [34]	Switzerland, university hospital	176 medical inpatients	Prospective cohort study on collaborative CLP screening	Referral rates increase from 4 to 32 %.
Jo et al. 2011 [36]	Korea, Seoul tertiary general hospitals	310 patients with suicide attempts	Questionnaire on patients	No significant difference in referral rate between history of suicide attempts and non-suicide attempters.
Pritchard 1972 [37]	UK, London general hospital	252 patients	Retrospective chart review of patients with psychiatric diagnosis	Patients with suicidal attempts have highest referral rates. Young age, functional psychosis associated with increased referral. Organic psychosis and previous psychiatric contact associated with decreased referral.
Caplan et al. 2008 [38]	US	N/A	Opinion piece	Work pressure suggested with increased CLP referrals
Wood & Bisson 2004 [39]	UK, maxillofacial surgery unit	58 patients with cancers of head and neck	Subjective reporting of experience with mental health nurse liaison	Increased referrals suggested with use of mental health nurse liaison.
Cohen-Cole et al. 1982 [40]	US, North Carolina Memorial Hospital	407 hospital doctors (34 % response rate)	37 item questionnaire	Physicians in internal medicine and family medicine tend to recognise patients with more psychological issues. Most hospital doctors are comfortable with managing psychiatric illness.
Lin et al. 2011 [52]	Taiwan, general hospital	111 obstetric and gynaecologic patients	Retrospective chart review	Low referral rates from obstetric and gynaecologic department.
Ni Mhaolain et al. 2008 [53]	Ireland, general hospital	96 surgical patients	Prospective evaluation of anxiety and depression in surgical patients	High prevalence of depression and anxiety in surgical patients.
Balestrieri et al. 2002 [54]	Italy, general hospital	1039 general inpatients	Cross sectional investigation of prevalence of depression among hospital patients	Identification of depression by hospital doctors in one third of all cases.
Fauman 1983 [55]	US, private urban hospital	265 hospital doctors (41.9 % response rate)	66 item questionnaire	Internists are more willing to refer suicidal attempts than surgeons. Internists are more willing to ask for consultations than surgeons for all other mental health conditions.
Hamdieh et al. 2015 [41]	Iran, Tehran, general hospital	300 non-psychiatric doctors (64.3 % response rate)	8 item questionnaire	Hospital doctors were more comfortable requesting for psychiatric consultations than managing psychiatric conditions themselves.
Jourdan & Glickman 1991 [57]	US, general hospital	380 psychiatric referrals	Retrospective chart review	High referral rates for determination of competency. Fear of medico-legal consequences and referrer’s anxiety suggested as reasons for referral rates.
Cepoiu et al. 2008 [42]	N/A	36 articles (comprising of 50935 inpatients)	Meta-analysis of recognition of depression in inpatients by non-psychiatric doctors	Sensitivity was found to be 36.4 % and specificity was 83.7 %
Clarke et al. 1995 [43]	Australia, Melbourne, Monash Medical Centre	987 medical and surgical patients	Prospective patient review, comparing diagnosis of depression made by psychiatrists and non-psychiatrists	Diagnostic concordance of depression 74 %, 41 % false positive rate and 15 % false negative rate.
Dilts et al. 2003 [44]	UK, York Hospital	346 medical inpatient consultations	Retrospective review comparing initial impression of primary medical providers to final psychiatric diagnosis	Initial diagnosis of cognitive disorders and substance use disorder is likely to be correct. Initial diagnosis of depression is wrong in half the cases.
Judd et al. 1997 [45]	Australia, Melbourne	392 HIV/AIDS patients referred for CLP	Retrospective chart review	Diagnostic concordance of depression was 79 %, 20 % false positive rate and 23 % false negative rate
Ryan et al. 1995 [46]	UK, Castle Hill Hospital	50 geriatric inpatients	Prospective cohort study examining agreement between psychiatrist and geriatricians on depression and dementia	Some evidence suggesting lower recognition of depression by geriatricians.
Yamada et al. 2012 [47]	Japan, Tokyo Metropolitan Geriatric Hospital	172 geriatric inpatients	Prospective diagnostic review of CLP consultations	Almost half of patients with depression diagnosed by referrers were found to be delirium.
Boland et al. 1996 [48]	US, teaching hospital	4396 inpatients referred for consultations	Retrospective chart review	40 % of patients initially identified by referrer to be depressed were found not to be depressed by psychiatry consultants
Canuto et al. 2015 [49]	Switzerland, University hospital of Geneva	148 inpatients over age of 60	Prospective cohort study	40 % of patients initially referred for depression were diagnosed with depression by CLP.
Smith et al. 1995 [50]	Australia, Melbourne, Monash Medical Centre	2347 inpatient referrals	Retrospective chart review	56 % of patients suspected by psychiatrist for substance use disorder were missed by referrers.
Su et al. 2011 [51]	Taiwan, region general hospital	1007 inpatient referrals	Retrospective chart review	Only 41.5 % of initial physician’s impression matches with psychiatrist’s final impression
De-Nour 1979 [56]	Israel	77 hospital doctors	Survey	Surgeons prefer consultation service, Physicians prefer liaison service.
Fenichel & Murphy 1985 [58]	US, Hospital of the University of Pennsylviania emergency department	12095 patients presenting to emergency department	Retrospective chart review	Patient with psychiatric history were associated with psychiatric referral
Marcus et al. 1987 [59]	US, 327 general hospitals	37221 patients with diabetes, 19484 patients with hip fractures, 25116 patients with COPD, 11770 patients with CABG	Retrospective chart review	Age negatively correlated to psychiatric consultation. Patients in urban settings are more likely to receive psychiatric consultation
Popkin et al. 1984 [60]	US	111 geriatric inpatients	Retrospective chart review	Compared to younger population, geriatric population was less often referred
Handrinos et al. 1998 [62]	Australia, Dandenong hospital	712 inpatient referrals	Retrospective chart review	Personality disorder predicts earlier referral. Depression predicts delayed referral.
Craig 1982 [63]	US, university hospital (not named)	362 inpatient referrals	Retrospective chart review	White patients are more likely to be referred. Once referred, there is no difference in care to white or non-white population.
Collins et al. 1992 [64]	US, San Diego Medical Centre	476 patients receiving psychiatric consultations compared with 14620 without psychiatric consultations	Retrospective chart review	Low referral rate for Hispanic patients.
Klein et al. 1996 [65]	US, Montefiore Medical Centre	48 inpatients	Survey	81 % agreeable to psychiatric consultation if their primary care doctor felt it was indicted

## Abbreviation

CLP: Consultation-Liaison Psychiatry
